# Easy axis anisotropy creating high contrast magnetic zones on magnetic tunnel junctions based molecular spintronics devices (MTJMSD)

**DOI:** 10.1038/s41598-022-09321-7

**Published:** 2022-04-06

**Authors:** Bishnu R. Dahal, Marzieh Savadkoohi, Andrew Grizzle, Christopher D’Angelo, Vincent Lamberti, Pawan Tyagi

**Affiliations:** 1 Center for Nanotechnology Research and Education, Mechanical Engineering, University of the District of Columbia, Washington, DC 20008 USA; 2 Y-12 National Security Complex, 301 Bear Creek Rd, Oak Ridge, TN 37830 USA

**Keywords:** Materials science, Nanoscience and technology, Physics

## Abstract

Magnetic tunnel junction-based molecular spintronics device (MTJMSD) may enable novel magnetic metamaterials by chemically bonding magnetic molecules and ferromagnets (FM) with a vast range of magnetic anisotropy. MTJMSD have experimentally shown intriguing microscopic phenomenon such as the development of highly contrasting magnetic phases on a ferromagnetic electrode at room temperature. This paper focuses on Monte Carlo Simulations (MCS) on MTJMSD to understand the potential mechanism and explore fundamental knowledge about the impact of magnetic anisotropy. The selection of MCS is based on our prior study showing the potential of MCS in explaining experimental results (Tyagi et al. in Nanotechnology 26:305602, 2015). In this paper, MCS is carried out on the 3D Heisenberg model of cross-junction-shaped MTJMSDs. Our research represents the experimentally studied cross-junction-shaped MTJMSD where paramagnetic molecules are covalently bonded between two FM electrodes along the exposed side edges of the magnetic tunnel junction (MTJ). We have studied atomistic MTJMSDs properties by simulating a wide range of easy-axis anisotropy for the case of experimentally observed predominant molecule-induced strong antiferromagnetic coupling. Our study focused on understanding the effect of anisotropy of the FM electrodes on the overall MTJMSDs at various temperatures. This study shows that the multiple domains of opposite spins start to appear on an FM electrode as the easy-axis anisotropy increases. Interestingly, MCS results resembled the experimentally observed highly contrasted magnetic zones on the ferromagnetic electrodes of MTJMSD. The magnetic phases with starkly different spins were observed around the molecular junction on the FM electrode with high anisotropy.

## Introduction

**Figure 1:**
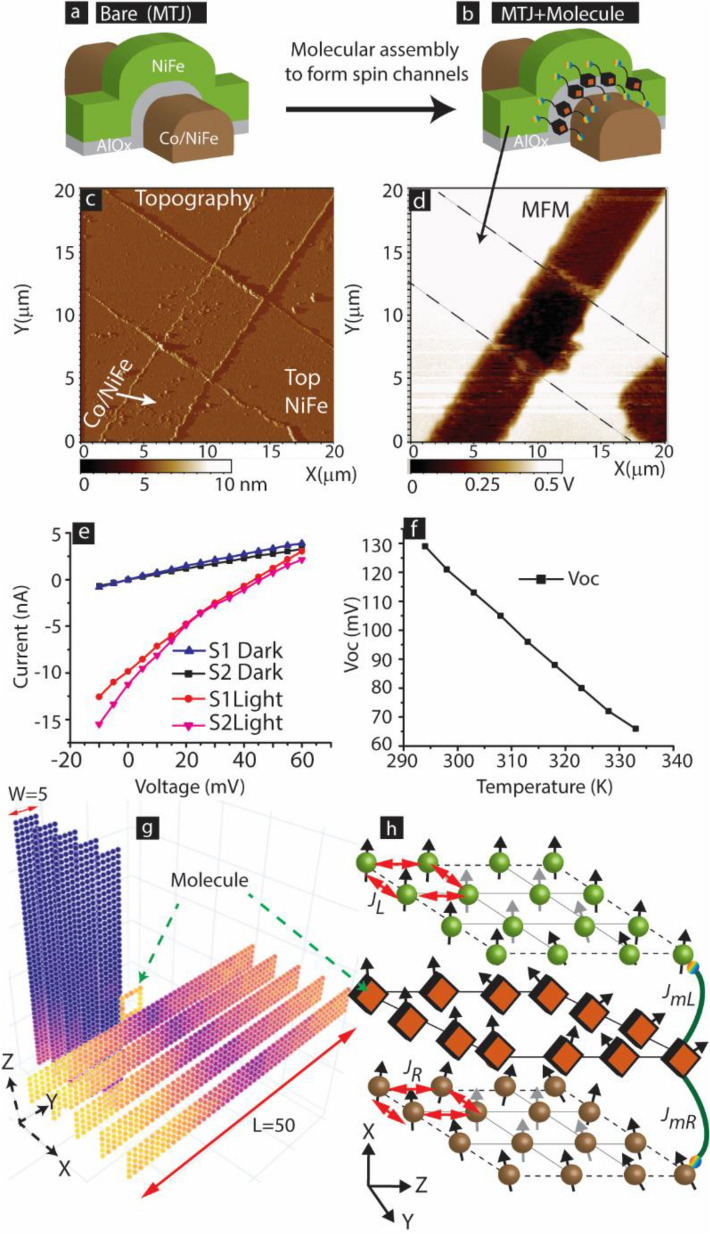
3D illustration of cross-junction shaped MTJ with exposed side edges (**a**) before and (**b**) after connecting paramagnetic molecules between ferromagnets. (**c**) AFM topography image of microscopic MTJMSD (**d**) showing the formation of strip on one magnetic electrode in MFM study. Dashed line are guides to eye for indicating impacted top NiFe region. Black color represents the higher end of the MTJMSD induced magnetic field, yielding a force on the MFM tip. On the other hand, the white color represents the lower bound of the magnetic force experienced by the MFM tip. MTJMSD showing (**e**) spin-photovoltaic effect in I–V study, and (**f**) temperature dependent open circuit voltage. (**g**) 3D atomic model of 5 × 5 molecular device analogous to conceptual MTJMSD illustration shown in panel (**b**). (**h**) Description of coupling energy around 4 × 4 molecular junction of 3D model shown in (**g**). (**g**) Is only for illustration purpose and include fewer molecules and interfacial FM atoms to present uncluttered view of exchange interactions.

Molecular spintronics devices (MSDs), utilizing electron spin property, can overcome the miniaturization and joul heating issues associated with the existing silicon-based devices technology^1^. Advantageously, MSDs^2^ may possess the tunable molecular spin states^3^ leading to the advancement of futuristic quantum computing relying on molecular magnets^4,5^. MSDs can also create synthetic antiferromagnetic materials resulting from the molecule-induced unprecedented strong exchange coupling^6,7^ between microscopic ferromagnetic (FM) electrodes, hence providing new frontiers for antiferromagnetic material-based devices^8–10^. Similar to commercially successful spin-valve devices, such as magnetic tunnel junctions(MTJs)^11,12^, MSDs based logic and memory devices are also expected to possess bistable or tunable multiple states^6,13–15^. Typically bistable states in MTJs are realized by the utilization of two magnetic electrodes of different magnetic hardness^16,17^; generally, magnetic electrodes are deposited by the sputtering process^18^. Different magnetic hardness in the MTJs is achieved by creating two multilayer electrodes with different magnetic anisotropies. MTJs soft magnetic layer switches spin direction with respect to rigid magnetic layer leading to bistable state^19^. However, in MSDs, experimental challenges have forced the utilization of nickel-like ferromagnet in source and drain electrodes^13,20^. The hard and soft magnetic layers of the MSD are created by changing the shape and thickness^13^ of nickel FM. Due to extreme fabrication challenges, the conventional nanogap junction approach was not able to mass-produce multilayer FM electrodes connected to molecular bridges^20,21^. To address the issue of using a full range of magnetic electrodes in MSDs, we utilized MTJ with exposed sides (Fig. 1a) as a testbed to produce a magnetic tunnel junction-based molecular spintronics device (MTJMSD). Several salient features and advantages of the MTJMSD approach compared to other conventional techniques are discussed in several reviews and related papers^2,22^.

For producing MTJMSD, molecular channels are bridged between two FM electrodes across the insulator of an MTJ (Fig. 1b). MTJMSDs have a multilayer structure in which two multilayers of FM electrodes are separated by a 2 nm insulator (Fig. 1a). The molecular nanostructure covalently bridged across MTJ’s insulator (Fig. 1b) can overcome the MTJ’s major challenges such as low spin coherence via tunneling barrier and high interfacial scattering effects at the ferromagnetic- insulator interfaces^23^. In our prior work, the paramagnetic molecules produced strong antiferromagnetic coupling between the FM electrodes of the MTJ testbed at room temperature^6,9^. In the prior experimental work, two FM electrodes possessed different magnetic hardness^16,24^. The molecule-induced strong antiferromagnetic coupling led to several novel phenomena such as several orders of magnitude current suppression at room temperature^9^, spin photovoltaic effects^25^, stark observations of different high contrasting magnetic phases^6^, and unstable yet several thousand percent changes in magneto-resistance^16^. Pasupathy et al.^20^ also showed intriguing Konodo resonance with Ni electrodes due to molecule induced strong coupling on break-junction form device. However, further advancement of MTJMSD requires a fundamental understanding of the impact of a large number of factors^26^.

There are various aspects that affect the overall magnetic properties of the MTJMSDs. Since MTJMSD is based on MTJ technology, hence FM electrode anisotropy^27^ will be highly critical in defining the switchable states for MTJMSD applications in memory devices. Among other factors^28^, anisotropy is also expected to produce unprecedented magnetic phases in MTJMSDs, opening a gateway for discovering novel magnetic metamaterials. Such MTJMSD may enable the realization of novel magnetic metamaterials by chemically bonding magnetic molecules and FMs with a vast magnetic anisotropy range. Since experimental studies cannot investigate the full range of permutations, this paper focuses on Monte Carlo Simulations (MCS). In this paper, MCS is carried out on the 3D Heisenberg model of MTJMSDs. We designed an MCS program to explore the magnetic properties of cross junction-shaped MTJMSDs; we opted for this shape to be consistent with experimentally studied MTJMSD geometry^6,9,16,25,29^. Our MCS study systematically varied the easy-axis anisotropy in one FM electrode and investigated the impact on the magnetic moment of MTJMSDs, magnetic susceptibility, and spatial correlation between molecules and FM electrodes.

## Experimental details and computational methods

The MCS study discussed in this paper is motivated by the experimental observation of magnetic zones formation on magnetic electrodes on MTJMSDs. We fabricated a Ta(~ 2 nm)/Co(~ 5 nm)/NiFe (~ 5 nm)/AlOx (2 nm)/NiFe (~ 10 nm) exposed edge cross-junction shaped MTJ (Fig. 1a,b). Paramagnetic Octametallic molecular clusters (OMCs)^30^ were covalently bonded to realize the device scheme shown in Fig. 1b. We have provided the extended details of the molecule attachment process in the prior publications^22,31^. Succinctly, we exposed MTJ testbed to OMC solution in dichloromethane solvent. Two external metal electrodes were immersed in the solution in the proximity of the intended MTJ area. Alternating ± 100 mV voltage was applied between the external metal electrodes to de-protect thiol groups present at the end of each alkane tether of OMC^30^. After the deprotection step, billions of OMC molecules became available to make the covalent bond with the metal layers on the MTJ stack. Many of these molecules bridged across the insulator of MTJ along the exposed side edges to form the conduction channel. With this approach, we were able to transform > 95% of the MTJ submerged in the OMC solution^24^. According to three independent magnetic studies, SQUID magnetometry, Ferromagnetic Resonance (FMR), and magnetic force microscopy (MFM), OMC paramagnetic molecules induced strong antiferromagnetic coupling between the two magnetic electrodes^24^. The in-depth discussion about magnetometry, FMR, and MFM is presented elsewhere^24^. Representative experimental data has been shown in the supplementary material for quick reference (Supplementary Material, Fig. S1). In general, ferromagnetic electrodes exhibit a certain degree of spin polarization^32,33^. However, molecular coupling transformed the common ferromagnetic electrode material into a highly spin-polarized material near junction area^9,25^. MTJMSD’s ferromagnetic electrodes settled into new magnetic states at room temperature due to the OMC-induced strong antiferromagnetic coupling and spin-filtering. In this state, ferromagnetic electrodes started exhibiting unprecedented spin-photovoltaic-like phenomenon^25^. The full details of experimental procedures are published elsewhere^6,25^.

We observed that a bare MTJ testbed without molecular channels exhibited uniform magnetic contrast in the MFM^6^. However, MTJ underwent a dramatic change at room temperature after bridging OMC channels. An MTJMSD that appeared continuous and in a sound state (Fig. 1c) exhibited the formation of starkly different magnetic phases around the junction area on the top magnetic electrode (Fig. 1d). It is counterintuitive that magnetic phases are formed on the NiFe top electrode when the bottom magnetic electrode with Co/NiFe bilayer films was more anisotropic than the top NiFe electrode. The observation of the magnetic contrast zones has remarkable significance in exploring the MTJMSD capabilities and various opportunities for the following reasons. (i) Appearance of high magnetic contrast provides vivid proof that OMC produced unprecedented strong exchange coupling between two microscopic ferromagnetic electrodes at room temperature. However, this observation implies that it will not be possible to easily move the magnetization direction of any of the two ferromagnetic electrodes by applying an external magnetic field due to molecule-induced strong exchange coupling. One needs to explore other combinations of molecule and ferromagnets to target switchability attributes in MTJMSDS. (ii) The observation of magnetic contrast provides direct evidence that OMC molecule channels can transform conventional NiFe-like ferromagnetic alloys into a new magnetic metamaterial. Our previous studies showed that OMC impacted ferromagnetic electrodes produced near 100% spin polarization^6,9,24^. Hence, the composite material that connects two ferromagnetic films with paramagnetic molecular bridges opens a new possibility of utilizing MTJMSD as the metamaterial. We recently published an observation of the intriguing spin-photovoltaic effect observed on MTJMSD at room temperature as one example of novel magneto-optical properties around the MTJMSD area^25^. Observation of high magnetic contrast regions around MTJMSD helped estimate the length scale of impacted ferromagnetic electrodes. (c) High magnetic contrast observation on soft NiFe ferromagnetic electrodes but not on the magnetically harder Co/NiFe bilayer bottom electrode suggests counterintuitive possibilities with MTJMSD testbed hinting towards the novel phenomenon that is beyond current understanding of conventional magnetic materials. (d) The width of the zone between two different magnetic phases indicates a dramatically abrupt transition. The abrupt transition was not observed in our prior MCS studies exploring the impact of variation in molecular spin state^34^, molecular coupling effect^35^, MTJMSD’s electrode thickness and length^36^, and competition of molecular coupling with the interaction via the tunnel barrier^37^. We hypothesized that the reason for the MFM data in Fig. 1d is associated with the strong spin filtering effect caused by the covalently bonded OMC channels^9^. We do not fully understand why the NiFe electrode is showing the contrasting magnetic phases. We also do not clearly understand the phase difference between the magnetic moment direction of the adjacent high contrasting areas. However, we do know for sure that OMC channels caused dramatic changes in the top NiFe electrode of MTJMSD. To investigate the science behind strip formation, we have explored several parameters involved in an MTJMSD, including molecular coupling strengths with ferromagnet, molecular spin state, metal electrode competing for molecule-defect coupling. We hypothesized if OMC created a new local magnetic anisotropy leading to the experimental observation of starkly different magnetic phases on a typical NiFe electrode.

According to prior literature, anisotropy on ferromagnetic electrodes can be due to chemical composition, shape, and external voltage^38^. Recently, it was shown that the application of ~ 100 mV electric field could change the relative occupation of the *3d*-orbital of the iron ferromagnetic electrode. The voltage-induced changes in the electron filling of *3d* orbitals were attributed to the change in magnetic anisotropy^38^. Interestingly, in MTJMSD, OMC induced strong antiferromagnetic coupling produced significant changes in spin polarization, i.e., electron filling of *3d* orbitals, leading to a built-in potential ~ 50–200 mV^25^. The proof of built-in potential is evident from the solar cell effect observed on the MTJMSD^25^. Current–voltage studies in the dark and light prove that an MTJMSD exhibited a solar cell effect (Fig. 1e). Extensive details of MTJMSD based spin-photovoltaic effect are published elsewhere^25^. This built-in potential was well stable above room temperature, though decreased linearly with temperature (Fig. 1f). We hypothesized that molecule-induced built-in potential, i.e., open-circuit voltage (*V*_*oc*_), creates voltage-induced anisotropy phenomena on MTJMSD electrodes, leading to contrasting magnetic phase formation (Fig. 1d). This paper explored the anisotropy factor with the motivation of understanding the potential cause of different phases separated by the abrupt boundaries.

Our MCS study investigates the effect of magnetic anisotropy on the equilibrium magnetic properties of the MTJMSDs. This study only focused on strong molecular coupling because we experimentally observed that ~ 10,000 paramagnetic molecules dramatically impacted the microscopic FM electrodes containing millions of atoms at room-temperature^9,25,29^. The range and value of different parameters are related to our previous experimental studies with OMC and the same thin-film configuration^24^, as studied here for the cross junction shaped MTJMSD. In our prior work^24^, OMC produced antiferromagnetic coupling with a strength of ~ 50% of the Curie thermal energy for NiFe electrodes. To make our MCS study relevant to the experimentally observed dominant type of molecule-induced antiferromagnetic exchange coupling^24^, we had focused on the case when molecules produced antiferromagnetic coupling with one FM electrode and ferromagnetic coupling with another FM electrode^9,25,29^. MTJMSD is represented by a Heisenberg model resembling the cross-junction-shaped MTJMSD (Fig. 1g). Based on prior experimental studies, showing that when atoms cluster size is more than 700 atoms, a ferromagnet behaves like bulk ferromagnet^39^, we included 1250 atoms in each FM electrode. For this task, we generally fixed FM electrode Heisenberg model dimensions to 5 × 5 × 50. The atomic details of the magnetic interactions in the Heisenberg model near molecule-FM electrode junction are shown in Fig. 1h. The Heisenberg coupling across the ferromagnetic atoms of left and right electrodes are represented by ${J}_{L}$ and ${J}_{R}.$ In this study, we fixed ${J}_{L}$ = ${J}_{R}$ = 1 to signifies the highest exchange coupling strength. It is noteworthy that these two exchange coupling parameters also define the thermal energy for the two FM electrodes to transition from ordered to disordered states^40,41^. In the classical Monte Carlo simulations, thermal energy at which ordered to disordered transition occurs is comparable to ${J}_{L}$ and ${J}_{R}$^40^.

Molecules’ antiferromagnetic coupling strength with the ferromagnetic atoms of the left electrodes and ferromagnetic coupling with the ferromagnetic atoms of right electrodes are represented by ${J}_{mL}$ and ${J}_{mR}$, respectively. According to our prior work in which we utilized 12 carbon long alkane tethers to connect OMC molecule core to FM electrodes. The order of magnitude of ${J}_{mL}$ and ${J}_{mR}$ was ~ 0.5 times Curie temperature of NiFe^24^. These two ${J}_{mL}$ and ${J}_{mR}$, parameters encompass the coupling strengths arising due to the different types of molecules used for linking paramagnetic molecules to FM electrodes. Hence, ${J}_{mL}$ and ${J}_{mR}$ represents cumulative coupling strength between FM electrodes and the paramagnetic core and can be stronger than that observed with OMCs we used in experimental work^30,42^. For example, a six atom long alkane tether may produce a much stronger coupling as compared to the 12 atoms long alkane tether to shorten the gap between FM electrodes and paramagnetic molecule core (Fig. 1h). Also, the rainbow color atom in Fig. 1b,h shows the sulfur-like atoms that covalently bond the molecules with the FM electrodes. Such molecule-NiFe bonding enables the strong mixing of metal and molecular energy levels. The chemist can produce OMCs like molecules with smaller alkane tethers^30,43^. Smaller tether molecule lengths allow ${J}_{mL}$ and ${J}_{mR}$ coupling strength increasing exponentially as compared to what we observed^24^. To encompass possibilities of stronger molecular exchange coupling, we fixed the magnitude of ${J}_{mL}$ and ${J}_{mR} to 1$. We hypothesize that the upper bound for ${J}_{mL}$ and ${J}_{mR}$ magnitude is the strength of exchange coupling within the FM electrodes which is set to 1.

Thermal energy was varied by changing *kT* parameter. For most of the studies, *kT* was fixed to be 0.1. The rationale for choosing *kT* = 0.1 was based on our prior experimental studies showing that OMC effect was stable well above room temperature. Assuming *kT* = 1 represent the Curie temperature of different FM electrode. For example, *kT* = 0.1 will be equivalent to ~ 60 °C for Ni^41^.

We have surmised that paramagnetic molecules^30,42^ are akin to a single unit that can be defined as an atomic analog (Fig. 1h). Our MCS program is capable of computing the effect of intramolecular spin interaction (*J*_*mol*_) and molecular anisotropy (*D*_*mol*_), as shown in Eq. (1). However, the following is the rationale for representing complex single-molecule magnets (SMM) with the atomic analog. (i) We successfully employed this approach in the prior MCS study to explain several experimental studies on MTJMSD^24,29^. (ii) Prior molecular device research has successfully employed generic analytical models to understand experimental data. For example, Simmons tunneling model^44^ was used to understand the transport characteristics through SMMs^9,29,31^. (iii) Molecules in the device form generally follow generic single-electron device physics^45^. Selzar et al.^45^ have shown that conventional quantum dot device physics can be employed to interpret molecular device data without delving into the atomic configuration of the molecules of interest. (iv) According to experimental data on powder form, SMMs generally settle in different spin states at different temperatures^30^. It is a tremendous challenge to conduct temperature-dependent simulations since DFT, like conventional approaches, only works for zero temperature. Additionally, micromagnetic simulations are limited and challenging in discretizing 1–10 nm molecular device elements and microscopic FM electrodes in complex MSD geometry^46^. (v) Experiments to identify the actual molecular spin state in a microscopic MTJMSD are exceptionally challenging due to the limitations of measurement techniques and the inability to reach the exposed side edge of MTJMSD. We do not claim that our approach of representing paramagnetic molecules in MCS simulation is perfect; however, it undoubtedly provides a pathway to simulate complex and microscopic MTJMSD one can test experimentally. We focused on the impact of the paramagnetic molecule by setting a fixed cumulative molecule spin state (*S*_*mol*_) to 1. We did not vary intra-molecule coupling (*J*_*mol*_) and anisotropy factor (*D*_*mol*_). In our recent MCS study, we found nature of molecule spin state impact was similar beyond a 0.2^34^. To make this study practical, we have fixed molecule and FM electrode spin state to 1, i.e., all *S*_*i*_ = 1. This FM electrode spin magnitude may be different for various types of FM electrode. However, we obtained good insights with the *S*_*i*_ = 1 in our recent MCS study related to MTJMSDs^34,37^.

We varied easy-axis anisotropy in one FM electrode while another FM electrode was isotropic during the simulation. We studied the impact of unidirectional easy-axis anisotropy along the y-direction ($${A}_{Ly})$$ of the left FM electrode. We varied *A*_*Ly*_ from minimum anisotropy ($${A}_{Ly}=0)$$ to maximum anisotropy ($${A}_{Ly}=1)$$ while keeping all other parameters fixed. We surmise that the upper bound of easy-axis anisotropy is the magnitude of exchange coupling strengths within the FM electrode. The MTJMSDs device energy was minimized to achieve stable energy state and spin states by performing 500 million iterations. The initial spin vector state of molecules and FM electrodes were set in a random direction. We generally preferred random states to ensure that all the stable state configuration results from simulation parameters are not due to metastable initial ordered states that may persist until the end of the simulation. MTJMSD stable spin states within electrodes and molecules were obtained after performing 500 million iterations through the following energy stabilization equation:

1 $$E=-{J}_{L}\left({\sum }_{i\in L}{\overrightarrow{S}}_{i}\cdot{\overrightarrow{S}}_{i+1}\right)-{J}_{R}\left({\sum }_{i\in R}{\overrightarrow{S}}_{i}\cdot{\overrightarrow{S}}_{i+1}\right)-{J}_{mL}\left({\sum }_{i\in L,i+1\in mol}{\overrightarrow{S}}_{i}\cdot{\overrightarrow{S}}_{i+1\left(mol\right)}\right)-{J}_{mR}\left({\sum }_{i-1\in mol,i\in R}{\overrightarrow{S}}_{i-1\left(mol\right)}\cdot{\overrightarrow{S}}_{i}\right)- {A}_{Ly}\left({\sum }_{i\in L}{\overrightarrow{S}}_{iy}^{2}\right)-{J}_{mol}\left({\sum }_{i-1\in mol,i\in R}{\overrightarrow{S}}_{i-1\left(mol\right)}\cdot{\overrightarrow{S}}_{i\left(mol\right)}\right)-{D}_{mol}\left({\sum }_{i\in L}{\overrightarrow{S}}_{i\left(mol\right)}^{2}\right),$$

where *S* represents the spin of individual atoms of FM electrodes and molecules in the form of 3D vectors. Magnetic properties of the device were quantified in terms of total magnetization of the MTJMSD, which is the sum of molecular magnetic moments magnetization of left and right ferromagnetic electrodes. Since the magnetization of the molecules is significantly small compared to the left and right FM electrodes, molecular magnetization does not impact the magnitude of MTJMSD magnetization in an equilibrium state. However, molecular bridges are the only medium that transfers the impact of variation in one electrode to another electrode. Extensive details about the MCS process are published elsewhere^24^. In this paper, we studied spatial correlations of the molecules to the ferromagnetic electrodes and the magnetic susceptibility of the MTJMSDs device to understand the magnetic phase transition on the electrodes. For keeping the discussion generic, the exchange coupling parameters, magnetic anisotropy, and thermal energy are the unitless parameters throughout this computational study.

## Result and discussions

**Figure 2:**
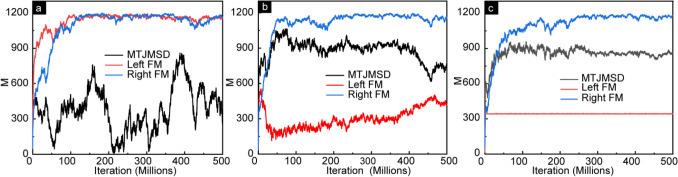
Magnetic moment vs. iteration counts showing temporal evolution of the MTJMSD, left FM, and right FM for the cases of (**a**) $A{L}_{Y}$= 0, (**b**) $A{L}_{Y}=0.1,$ and (**c**) $A{L}_{Y}=1.$ In all the cases *kT* = 0.1. *J*_*mL*_ =  − 1 and *J*_*mR*_ = 1, S = 1for molecules and FM electrodes. MTJMSD started from random state and stabilized over 500 M iterations.

To study the impact of anisotropy on the evolution of equilibrium magnetic properties of MTJMSD, we conducted the temporal progression (time vs. magnetization) studies of MTJMSD for different ${A}_{LY}$ (Fig. 2). We represent time as the iteration counts in MCS. Based on the MTJMSD Heisenberg Model, left-FM and right-FM electrodes can attain the maximum magnitude of the magnetic moment of 1250. At the same time, MTJMSD’s maximum magnetic moment can settle around 2516 (1250 for each FM electrode and 16 for molecules). MTJMSD maximum magnetic moment is possible when spins of all the molecules, the left and right FM electrodes’ are ideally aligned in the same direction. For the case of ${A}_{Ly}=0$, MTJMSD magnetic moment is expected to dominate the molecule-induced antiferromagnetic coupling. Under the molecular coupling effect, MTJMSD magnetic moment is expected to settle near-zero magnetic moment. Indeed, time vs. magnetic moment data for ${A}_{Ly}=0$, the total magnetic moment of the MTJMSD is close to zero, see Fig. 2a. It is noteworthy that the left and right FM-electrodes have an equal magnitude of the magnetic moment, but most of the magnetic spins are antiparallel. In Fig. 2a, the magnetic moment of left, right, and overall MTJMSDs are represented by Left-FM, Right-FM, and MTJMSD, respectively. In this state, MTJMSD’s magnetic moment settled to fluctuating low values (Fig. 2a). The fluctuating low MTJMSD’s magnetic moment as compared to the individual FM electrodes signifies the possibilities of various meta-stable FM electrode configurations. Our previous work observed that MTJMSD’s electrodes could settle in single or multiple phases^35,36^. Energetically, these states are pretty similar in energy. Hence, the final MTJMSD magnetic moment results from the type of magnetic phase stabilizing on ferromagnetic electrode^35,36^.

**Figure 3:**
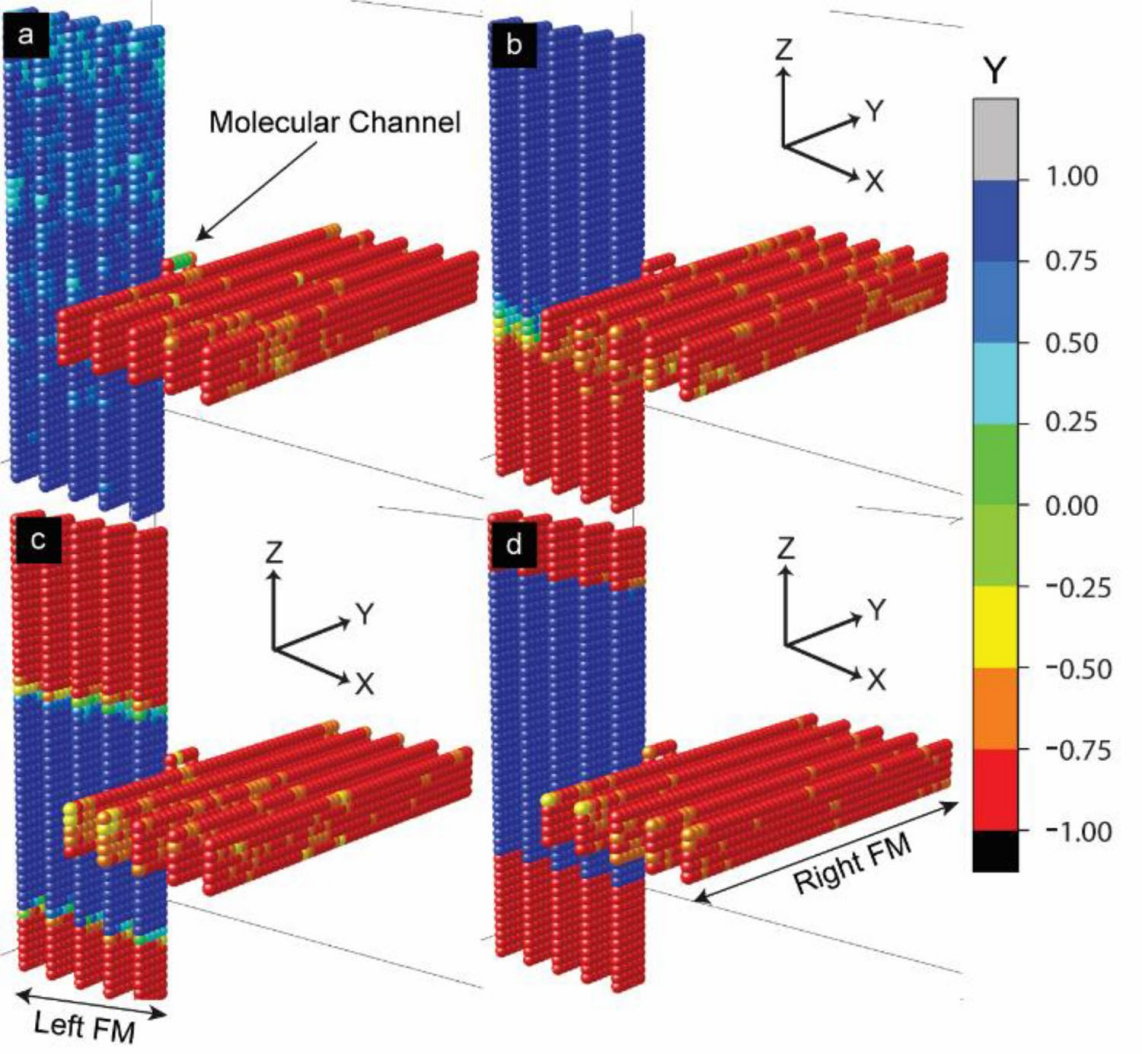
Simulated 3D lattice model of the MTJMSD measured at $kT$ = 0.1. (**a**) For ${A}_{LY}$ = 0 direcrtion of stablization can be anywhere in 3D space. For (**b**) ${A}_{LY}$ = 0.1, (**c**) ${A}_{ALY}$ = 0.4, and (**d**) ${A}_{LY}$ = 1 electrode spins predominently align parallel or antiparallel to the easy Y axis. For (**a**) color bar represent magnetic moment along the direction of stablization that is not necessarily easy axis. For (**b–d**) color bar represent magnitude of magnetic moment parallel or antiparallel with respect to Y direction.

Experimental studies have evidenced the realization of paramagnetic molecule-induced exchange coupling leading to a very low magnetization state (antiparallel FM electrodes)^6,24^. MTJMSD’s low magnetic moment provided a plausible explanation for the observed—six orders of magnitude current suppression phenomenon at room temperature^9^. We explored the role low anisotropy energy ${A}_{Ly}$ as compared to molecular exchange coupling strength. We observed that when anisotropy in left electrode was around 10% of molecular coupling strength, i.e. 1, the left electrode started exhibiting starkly opposite magnetic regions (Fig. 2b). When ${A}_{LY}= 0.1$, the anisotropy is forcing to align the magnetic spins of the left FM electrode to a particular orientation that is causing the overall FM electrode magnetic moment to be much lower (Fig. 2b) compared to the case when anisotropy was zero (Fig. 2a). It appears that anisotropy is creating domains of oppositely aligned spins that cancel a part of the left FM electrode moment leaving significantly less net magnetic moment (Fig. 2b). In this case, the MTJMSD magnetic moment was dominated by the right electrode magnetic moment (Fig. 2b). MTJMSD magnetic moment for weak anisotropy was less unstable as compared to the case of zero anisotropy (Fig. 2a). As shown in Fig. 3, increasing anisotropy produces oppositely aligned phases of variable length, producing some degree of variation in different runs. In the subsequent simulations, we observed that increasing easy-axis anisotropy appears to stabilize MTJMSD. For the ${A}_{LY}=1$ case (Fig. 2c), the total magnetization of the device is significantly stable compared to the case when ${A}_{LY}= 0$. When ${A}_{LY}=1$, magnetic moment of the left FM electrode fixed to an unwavering constant saturated magnetic moment right from the beginning. Since molecular analogs are forcing FM electrodes to be antiparallel, the net magnetic moment of the MTJMSD is a difference of the magnetic moment of the left and right FM electrodes (Fig. 2c). In Fig. 2b,c, we observed left FM electrodes’ magnetic moment decreasing sharply with increasing anisotropy in this electrode.

To visualize the actual spin configurations of the left FM electrode and overall MTJMSD, we analyzed the atomic scale equilibrium moment of MTJMSD’s Heisenberg model, Fig. 3. In 3D atomic-scale representation, the left FM electrodes are represented by vertical lattices, while horizontal lattices represent right FM electrodes. In this model, molecules are represented as a small square between left and right FM electrodes. It is noteworthy that in the absence of magnetic anisotropy, the MTJMSD spin states can settle in any direction in 3D space^47^ (Fig. 3a). However, the application of ${A}_{LY}$ forced the MTJMSD’s spin states to be settled along Y-direction only. It means the magnetic moment of the two FM electrodes and molecules stabilized in parallel or antiparallel direction with respect to the direction of anisotropy, i.e., Y direction. Therefore, we have only presented Y-direction spin sates of MTJMSDs’ magnetic moment in Fig. 3b–d. When ${A}_{LY}=0,$ left FM electrode, right FM electrode, and bridging molecules have random spin states along the Y-direction. However, the spin states of the left FM electrode are closely opposite to that of the right FM electrode due to the antiferromagnetic coupling between left and right FM electrodes via the molecules, but the direction of MTJMSD stabilization can be anywhere, including Y (Fig. 3a). The case of MTJMSD equilibrium state without anisotropy is discussed elsewhere^47^. As we started increasing easy-axis anisotropy along Y-direction, we saw multiple magnetic phases within the left FM electrode. For ${A}_{LY}$ = 0.1, a diverse domain structure of spins with two extreme magnitudes started to appear on the left FM electrode. This domain structure represents the magnetic phase transition from one spin state to another and is responsible for the overall magnetic properties of the MTJMSD device and left FM electrode (Fig. 3b). As the magnitude of anisotropies increased to ${A}_{LY}$ $\ge$ 0.3, multiple magnetic domains of opposite spins started to appear on the left FM electrode, as shown in Fig. 3c for ${A}_{LY}$ $=$ 0.4 and Fig. 3d for ${A}_{LY}$ $=$ 1. The size and colors of these domains depended upon the magnitude of the anisotropy. Interestingly, the higher magnitude of anisotropy along the left FM electrodes could align the spin directions of molecules and atoms of right FM electrodes to a particular direction, as shown in Fig. 3c,d. Our study suggests that the impact of left electrode anisotropy is transferred to the right ferromagnetic electrodes through the molecular channels when the anisotropy is high. However, the direction of spin alignment for molecules and atoms of the right FM electrodes is not consistent on various simulation trials (Supplementary Material-Fig. S2). We surmise that several competing equilibrium states are possible and lead to different types of MTJMSD configurations.

**Figure 4:**
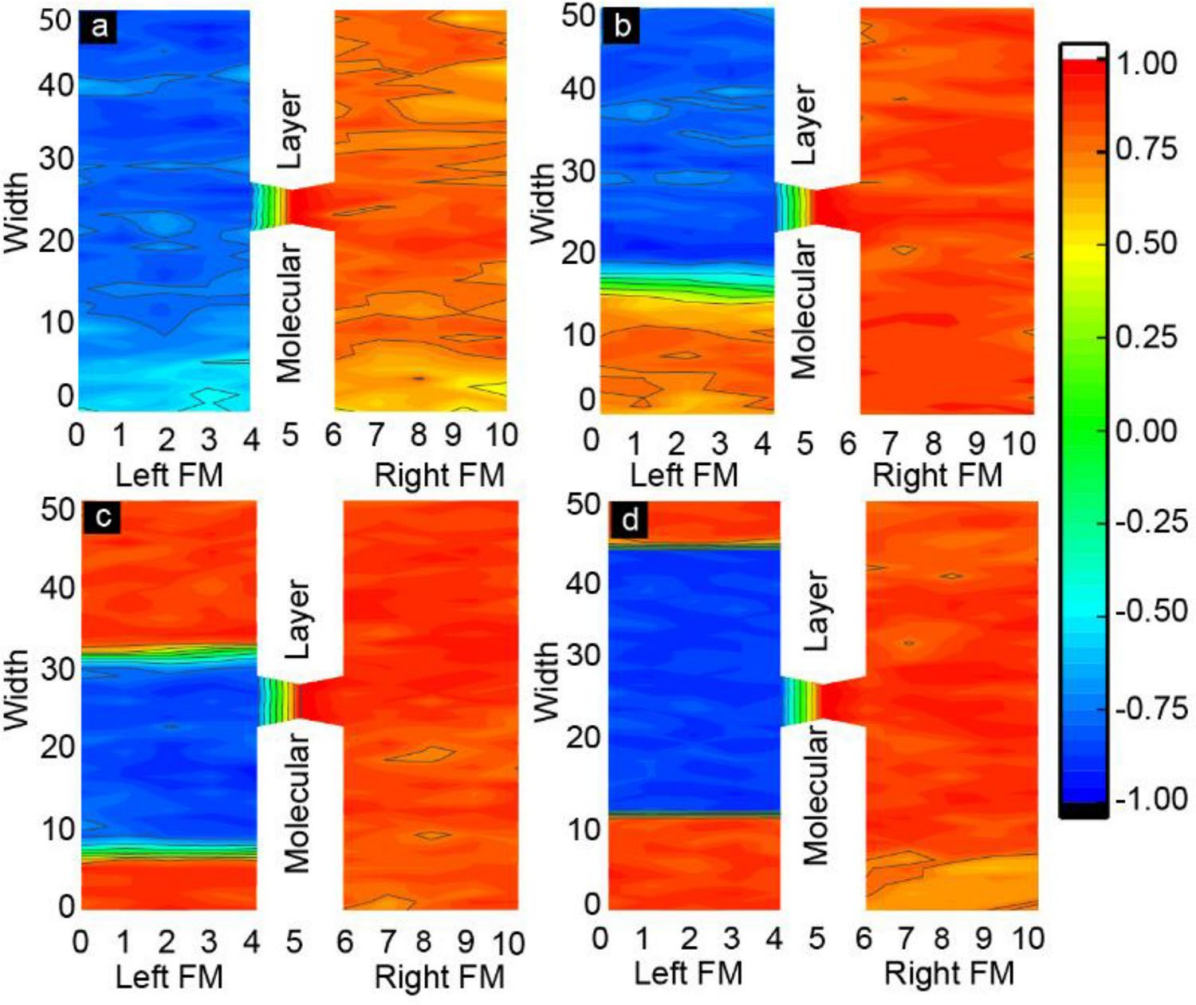
Molecule correlated FM electrode phases shown in the spatial-correlation factor contour plots of MTJMSD. SC is plotted for right and left FM electrodes and the magnetic molecule when (**a**) ${A}_{LY}$ = 0, (**b**) ${A}_{LY}$ = 0.1, (**c**) ${A}_{LY}$ = 0.4 and (**d**) ${A}_{LY}$ =1. (**d**) Is for Aly 1 or 0.4.

We further investigated the length scale of different phases in FM electrodes and spatial correlation between molecular spins and FM electrodes (Fig. 4). This spatial correlation study utilized stable state MTJMSD’s magnetic moment recorded after 500 million iterations from magnetic moment vs. iteration studies**.** To quantify the correlation of spins between molecules and atoms in different layers of the ferromagnetic electrodes in the presence of anisotropy, we have studied the customized spatial correlation factor (*SC*). *SC* is the vector dot product between the average molecular spin and the average of spins in each atomic row of two ferromagnetic electrodes. The equation used to calculate the *SC* is as follow:

2 $$\mathrm{S}C={ (S}_{m}\overrightarrow{x}+{ S}_{m}\overrightarrow{y}+{ S}_{m}\overrightarrow{z})\cdot{ (S}_{FM}\overrightarrow{x}+{ S}_{FM}\overrightarrow{y}+{ S}_{FM}\overrightarrow{z}).$$

**Figure 5:**
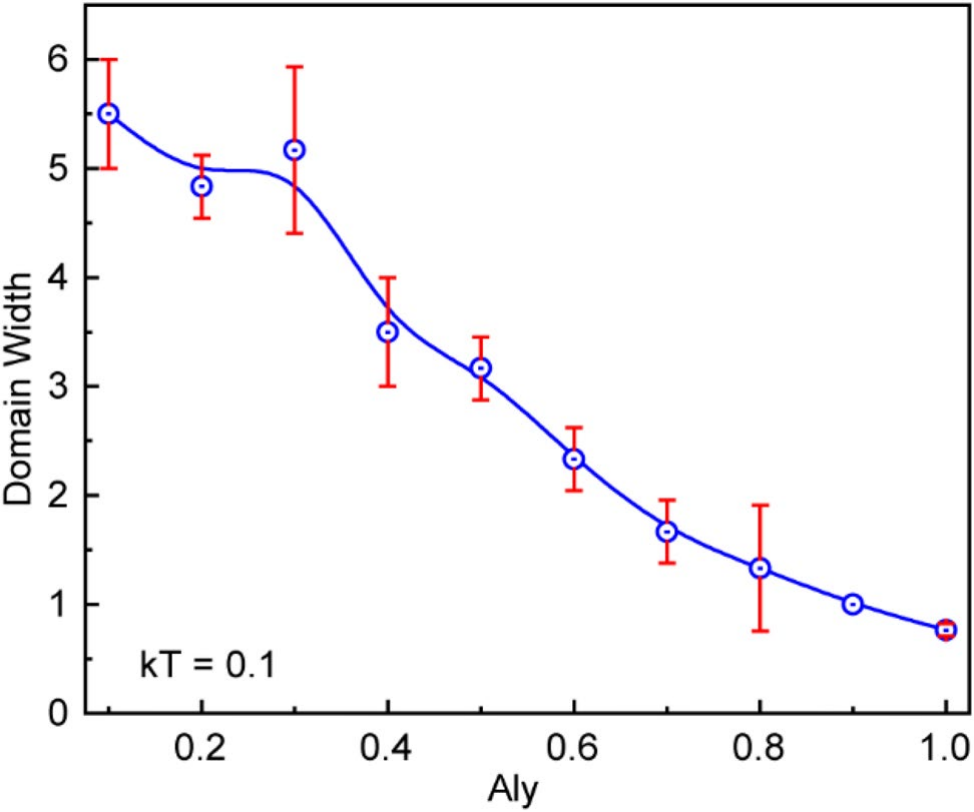
Width of the domain wall between two zones containing opposite magnetic spins as a function of easy-axis anisotropy (${A}_{LY}$) at $kT$= 0.1.

Here, $${S}_{m}\overrightarrow{x}, {S}_{m}\overrightarrow{y},$$ and $${S}_{m}\overrightarrow{z}$$ are the average spin vectors of molecules along x, y, and z direction, respectively. Similarly, $${S}_{FM}\overrightarrow{x}, {S}_{FM}\overrightarrow{y},$$ and $${S}_{FM}\overrightarrow{z}$$ are the spin vectors along x, y, and z directions, respectively. Positive *SC* represents the parallel alignment of the FM electrode layer with respect to molecules. Negative *SC* represents the antiparallel alignment of FM electrode layers and molecular layers. The magnitude of *SC* suggests the strength of correlation between molecule and FM electrode layers. The SC contours shown in Fig. 4 correspond to the cases of anisotropy shown in 3D lattice plots Fig. 3. Here, Fig. 4a is for $${A}_{LY}$$ = 0, Fig. 4b for $${A}_{LY}$$ = 0.1, Fig. 4c for $${A}_{LY}$$ = 0.4, and Fig. 4d for $${A}_{LY}$$ = 1. When $${A}_{LY}$$ = 0, the spin states of two FM electrodes are highly correlated with the spin states of the molecules. Molecule-induced strong antiferromagnetic coupling forced left FM and right FM electrodes to assume antiparallel states (Fig. 4a). Atomic Spins of left ferromagnetic atoms were negatively correlated with the molecular spins, while atomic spins of right ferromagnetic electrodes were positively correlated with the molecular spins. These correlations were expected in the MTJMSD Heisenberg model because molecules were antiferromagnetically and ferromagnetically coupled with left and right FM electrodes, respectively. Interestingly, the SC was high for the regions near molecules/FM junctions. Molecules tend to align their spins in strong correlation with the spins of FM electrodes in close proximity. Therefore, SC is typically higher near junction regions (Fig. 4a). With the application of small magnitude of anisotropy ($${A}_{LY}$$ = 0.1) a diverse domain structure of spins with two extreme magnitudes started to appear on the left FM electrode (Fig. 4b). From 0 to 15 atomic layers, molecule spins are positively correlated with the spins of the left FM electrode with the maximum SC of 0.8. We also investigated the boundary region width between two phases in the left -FM electrode using an SC contour plot. The maximum SC of 0.6 appeared around the 15th atomic row of the left FM electrode. Above the 19th atomic layer of the left FM electrodes, SC became negatively correlated with respect to molecular spin. When $${A}_{LY}$$ $$\ge$$ 0.3, multiple pockets of different spins orientations were observed within the left FM electrode. Interestingly, the maximum magnitude of SC was observed close to the transition zone. SC around 7th and 31st atomic width were 0.8 and 0.9, respectively, as shown in Fig. 4c. The magnetic phase transition occurs after the 7th and the 31st atomic position of the left FM electrode, Fig. 4c. As the magnitude of the easy-axis anisotropy increased above 0.3, the size of the stripes on the left FM electrodes became different (Fig. 4c) compared to weak anisotropy cases. Interestingly, the boundary region between two oppositely correlated phases became sharper (Fig. 4c). For $${A}_{LY}$$ $$=$$ 1, a big domain of negatively correlated atomic spins appeared, as shown in Fig. 4d. The negatively correlated big domain on the left FM electrode started at the 12th atomic position and persisted up to the 43rd atomic position, in this particular case. It is important to note that the domain wall width between two magnetic phases is also affected as the easy-axis anisotropy changes. In summary, increasing easy-axis anisotropy produced sharper domain boundary between high contrast magnetic zones. The gap between two domain boundaries appears to reduce with increasing easy-axis anisotropy (Fig. S5, Supplementary Section). The variation of domain width at $$kT$$ = 0.1 as a function of $${A}_{LY}$$ measured in terms of atomic layer thickness is shown in Fig. 5**.** The domain wall shown in Fig. 5 is the average of three trials of simulation, and the vertical error bar represents the standard deviation for these trials. The domain wall width kept decreasing with increasing $${A}_{LY}$$. The minimum value of the domain wall is at the maximum value of anisotropy confirms the sharpest magnetic phase transformation always occurs at the maximum $${A}_{LY}$$ ($${A}_{LY} = 1).$$

Currently, we are unable to provide the exact mechanism behind the evolution of contrasting domain/stripe forming due to increasing easy-axis anisotropy. Observing multiple domains with antiparallel spins and sharp domain walls resembles the Bloch wall phenomenon. Bloch walls of different widths are observed in many ferromagnetic materials due to anisotropy^41^. According to prior literature, the multi-magnetic phases develop when the material possesses a hard magnetic phase with the high value of the coercive field and the low magnetic phase with the low coercive fields^48^. These phases mostly had opposite magnetic spins and competed against each other, keeping the total magnetization of the device low. Based on prior literature, we hypothesize that the appearance of any form of anisotropy is a potential cause of magnetic domain formation^49^. The magnetic domain’s width and thickness of the transition region or domain wall depend on the specific type of anisotropy and its competition with exchange energy^50^. In a previous MCS study without any anisotropy ($${A}_{LY}=0),$$ we observed that as length of FM electrodes increased the left and right FM electrode start exhibiting multiple diffusive domains or stripes with a variety of stable spin direction^36^. Interestingly, the transition zones or walls between two domains were arbitrarily wide and diffusive. According to prior literature^41^, multiple domain formation is generally a consequence of increasing anisotropy. It is noteworthy that different molecular spin states in the MTJMSD Heisenberg model may produce local anisotropy at the FM-molecule-FM interface producing one potential cause of diffusive domain wall and contrasting zones seen with longer FM electrodes generally^36^. In this paper, we observed the increasing easy-axis anisotropy forced the appearance of a magnetic domain in much smaller 50 atom long FM electrodes, as discussed in Figs. 3, 4 and 5. Easy axis anisotropy in the left electrode also created a global anisotropy in the whole MTJMSD 3D model due to the strong molecular coupling. When the left FM electrode had anisotropy, the right FM electrode without any isotropy exhibited different magnetization properties since molecules connect the two electrodes antiferromagnetically. Figure 4a–d clearly shows that the right electrode exhibited a stronger and uniform correlation with molecules with increasing anisotropy in the left FM electrode. Further work is needed for developing clear understanding of mechanisms.

**Figure 6:**
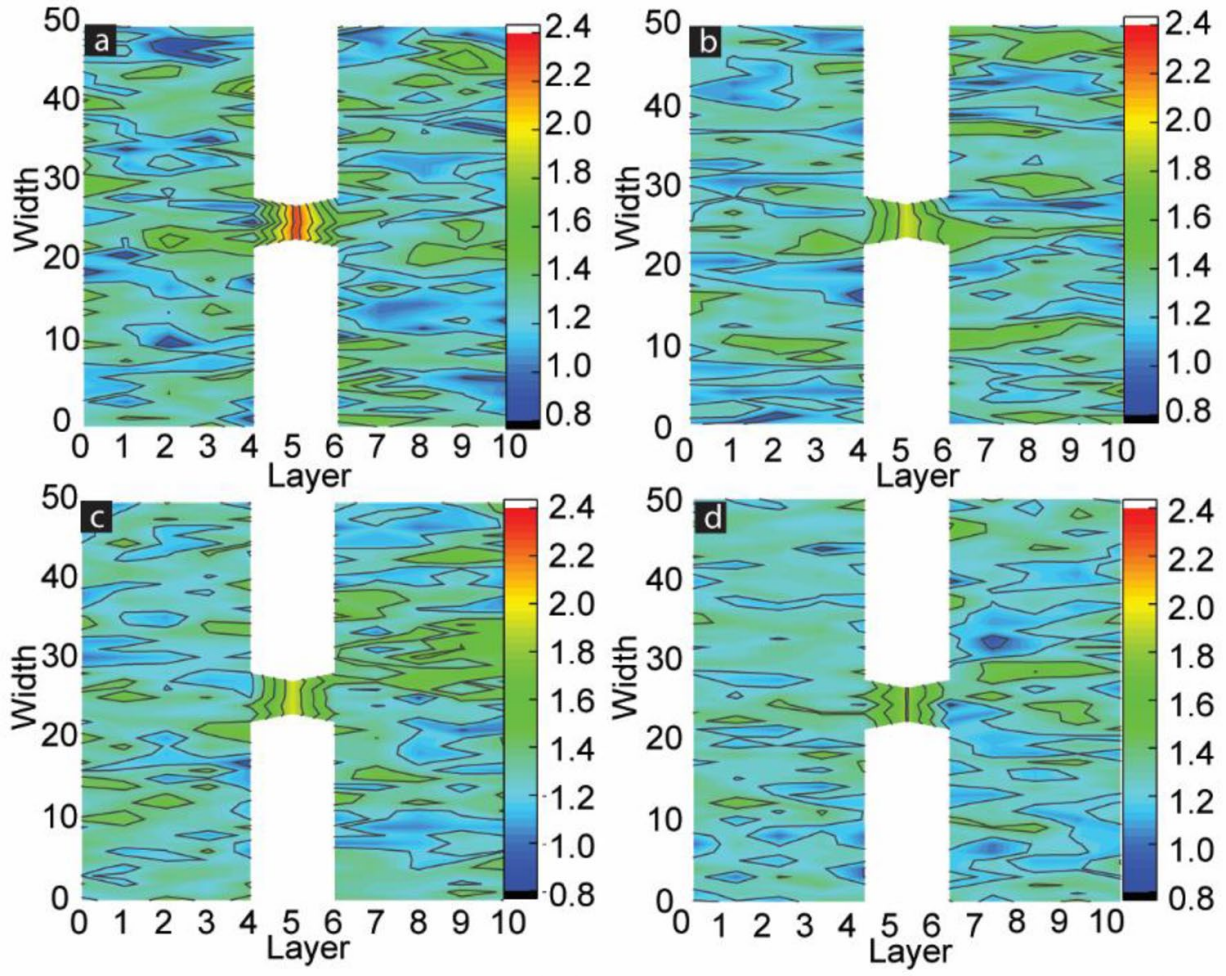
Contour plot of Magnetic susceptibility of MTJMSD with (**a**)${A}_{LY} = 0$, (**b**) ${A}_{LY} = 0.1$, (**c**) ${A}_{LY} = 0.4$, and (**d**) ${A}_{LY} = 1$. No external magnetic field was applied.

To understand how the MTJMSDs will behave with the external magnetic field, we calculated the spatial magnetic susceptibility in the different sections of the MTJMSD, as shown in Fig. 6. Spatial magnetic susceptibility $({\chi }_{s})$ was calculated by considering the group of atoms present along the width of the electrodes, i.e., shorter dimension parallel to the molecular plane (Fig. 3) using Eq. (3). Equation (3) is based on a well-established procedure specific to Monte Carlo simulation^40^ and did not require an external magnetic field for the computation.

3 $${\chi }_{s}=\frac{\chi }{{k}_{B}}=\frac{1}{{k}_{B}T}\left(\langle {M}^{2}\rangle -{\langle M\rangle }^{2}\right).$$

When $${A}_{LY} = 0$$, Fig. 6a, it was observed that the molecules are several times more susceptible to the external magnetic field as compared to the left and right FM electrodes (Fig. 6a). Hence, the MTJMSDs will behave differently to the external fields in the molecular regions and FM electrode regions. With the application of small magnitude of anisotropy ($${A}_{LY} = 0.1)$$ (Fig. 6b), molecules are slightly less susceptible compared to the case of no anisotropy (Fig. 6a). But, at the same time, FM electrodes are somewhat brighter than the no-anisotropy case (Fig. 6b). The magnetic susceptibility of the two phases on the left-FM electrodes is statistically the same as we could not see two different regions in the susceptibility plot (Fig. 6b). The same observation is continued until we reached high anisotropy (Fig. 6c,d). The susceptibility of the FM electrodes has not changed significantly with the application of easy-axis anisotropy, but the paramagnetic molecules are observed to be less susceptible to the application of the anisotropy as it is decreased from 2.2 to 1.3 for $$A{L}_{y}$$ = 0 to 1, respectively**.** We realize a need for in-depth experimental MTJMSD studies investigating the role of variation in the plane and out-of-plane anisotropies in the presence of various types of molecular device channels. Such study will provide foundational work to apply MTJMSD as a spin valve or STT-RAM devices^50^.

**Figure 7:**
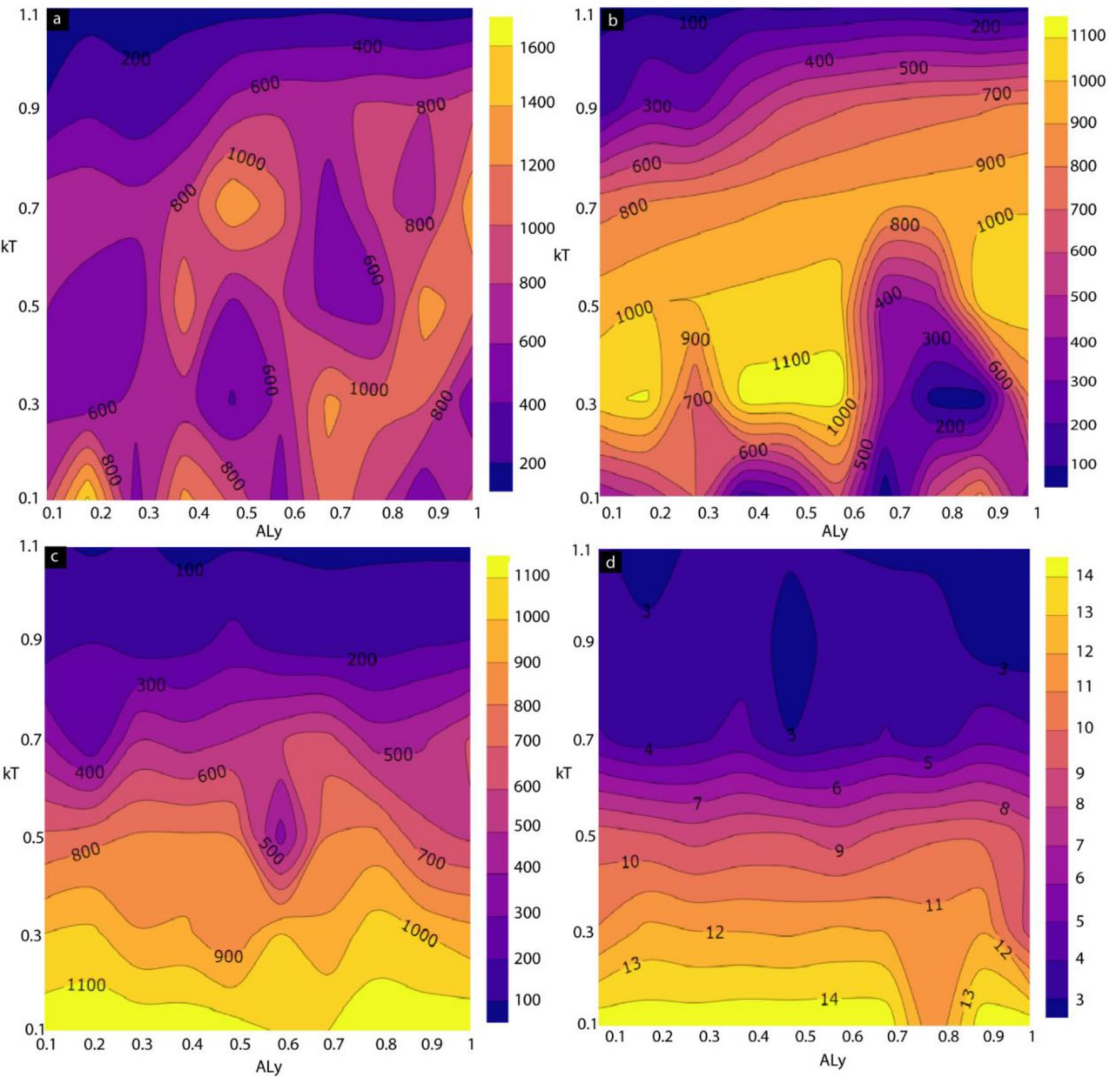
Magnetic moment of MTJMSD and its components was studied as function of thermal energy and easy axis anisotropy strength. Contour plot of (**a**) MTJMSD, (**b**) left FM electrode and (**c**) right FM electrode, and (**d**) molecular magnetic moment, as a function of in-plane anisotropy along y-spin direction and thermal energy ($kT$).

Thermal energy plays a critical role in defining the magnetic properties of ferromagnetic materials. To study the magnetic behavior of the device at higher thermal energy, we studied the temperature dependence of the magnetic properties of the full MTJMSD and its components. For this study, we varied thermal energy (*kT*) from 0.1 to 1.1. Figure 7 shows the contour plot for the magnetization of the MTJMSD (Fig. 7a), left FM electrode (Fig. 7b), right FM-electrode (Fig. 7c), and molecules (Fig. 7d) as a function of $kT$ and ${A}_{LY.}$ Overall, device magnetization is determined by adding the magnetic moment of the molecule, left FM electrode, and right FM electrode (Fig. 7a). It is noteworthy that alternating high and low magnetic moment phases appeared as anisotropy for a low-temperature regime. For *kT* ~ 0.3, MTJMSD settled in different phases of total magnetic moment varying from ~ 400 to ~ 1400 range (Fig. 7a). It is critical to analyze individual magnetization of left-FM, right-FM, and molecules at various $kT$ and anisotropies values to understand the MTJMSD data in Fig. 7a. Figure 7b shows the magnetization of the left FM electrodes. The highest value of magnetization ($\sim 1150)$ was observed when the thermal energy was around 0.3. For $kT\le$ 0.3, multiple magnetic phases have developed. As $kT$ increased beyond ~ 0.5, these magnetic phases started to vanish (Fig. 7b). However, the magnetization of the MTJMSD device would be higher due to the presence of anisotropy on the left FM electrodes and increasing randomness due to increasing *kT* (Fig. 7a). As a result, for $kT\ge 0.9,$ the magnetization of the overall MTJMSDs is closely equal to that of the left FM electrodes since the easy-axis anisotropy is only present on the left FM electrode. When $kT\ge 0.7$, the magnetization of the overall MTJMSDs drop due to the dominating effect of thermal agitation impact on the right FM electrodes (Fig. 7c). The impact of $kT$ on the right FM electrode without anisotropy (Fig. 7c) was much more pronounced. Consistent with the data shown in Figs. 4 and 6, magnetization of the right FM electrode did not show the appearance of pronounced phases (Fig. 7c). It is quite clear that anisotropy is a significant factor in causing the appearance of phases in the left FM electrode (Fig. 7b) and on MTJMSD (Fig. 7a). We also studied the molecule behavior with $kT$ and anisotropy in the left FM electrode. Molecule magnetic moment remained high at low temperature.

Interestingly, around *A*_*Ly*_ = 0.8 molecules magnetic moment was reduced from 14 to 11 (Fig. 7d). This result suggests that anisotropy in one electrode may influence the paramagnetic molecular device elements. The impact of anisotropy on molecules leads to a significant change in the MTJMSD transport properties. Also, it is important to note that for weaker anisotropy values, molecules remained unaffected (Fig. 7d). Figure 7c,d show the magnetization of the right FM electrode and molecules, respectively. The corresponding 3D plots of these contour plots have presented in the supplementary data (Supplementary Material-Fig. S3).

**Figure 8:**
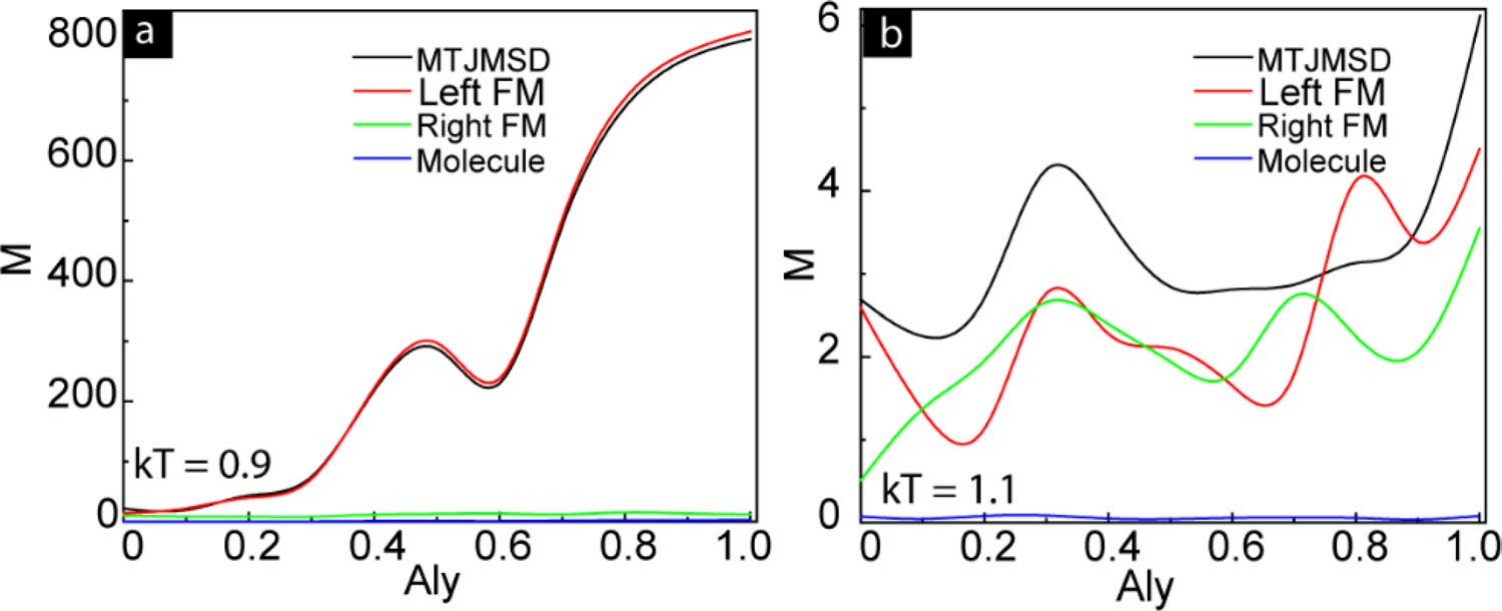
Anisotropy dependence of magnetic moment of MTJMSD, left FM, right-FM, and molecules at (**a**) *kT* = 0.9 and (**b**) *kT* = 1.1.

We also investigated the impact of anisotropy on the MTJMSD properties near the curie temperature. Figure 8 shows the magnetization as a function of anisotropy measured at constant thermal energy $kT$. Here, we present the anisotropy dependence of magnetization just below and above the Curie temperature of the device, i.e., $kT$ = 0.9 and $kT$ = 1.1. Figure 8a shows that the presence of easy-axis anisotropy on the left FM electrode keeps the high value of magnetization of MTJMSD at *kT* = 0.9. When the temperature is reaching close to the Curie temperature ($kT$ = 0.9), the thermal agitation overcomes the effect of the Heisenberg couplings present on the right FM electrodes and molecules. Therefore, the magnetization of the right FM electrode and molecules became bearly zero, Fig. 8a. The magnetization of the left FM electrode depends on the anisotropy and having the maximum anisotropy at $A{l}_{y}$ = 1. MTJMSD’s magnetization was governed by the left-FM electrode that could survive at high thermal energy (Fig. 8a). As the temperature increased above the Curie temperature total magnetization of MTJMSD is settled close to zero due to higher thermal fluctuation. The distribution of the magnetic spins on the left and right FM electrodes was completely random, as shown in Fig. 8b. This observation is inconsistent on every iterations trial, as shown in Supplementary Fig. S4. This study suggests that designing an MTJMSD for high-temperature applications should include ferromagnetic electrodes with high magnetic anisotropy. We are unsure if high anisotropy may also help against radiation hardening in space and nuclear environments.

## Conclusions

We have systematically studied magnetic tunnel junction-based molecular spintronics devices (MTJMSDs). The MTJMSDs were computationally simulated using Monte Carlo Simulation (MCS). These computational studies were motivated by previously fabricated and experimentally studied cross junction shaped MTJMSD by our group. We observed that molecule-induced strong exchange coupling produced highly contrasting magnetic phases on the ferromagnetic electrodes around MTJMSD. MCS study in this paper showed that variation in anisotropy of ferromagnetic electrodes produced highly contrasting magnetic phases on a ferromagnetic electrode in an MTJMSD. Our study revealed that the magnetization of the overall device was decreasing up to the thermal energy $kT\le 0.3$ due to the competing effect of multi-magnetic phases of opposite spins. The easy-axis magnetic anisotropy can hold the higher values of overall magnetization of the device despite the presence of higher thermal fluctuation. But the magnetization of the device was started to decrease abruptly for $kT\ge 07.$ Since the applied magnetic easy-axis anisotropy was present only on the left ferromagnetic electrode, the overall magnetization of the device at a higher temperature was governed by the left ferromagnetic electrode. Future studies will investigate MTJMSD design containing both FM electrodes with different degrees of magnetic anisotropy. Such studies are expected to yield advanced molecular devices for high-temperature applications and may be suitable for space and nuclear environments where significant radiation may be present. The width of the transition region between two oppositely oriented domains decreased with increasing anisotropy strength. The anisotropy-induced magnetic phases governed the equilibrium state magnetization of the MTJMSD. The anisotropy played a critical role in producing highly stable magnetic phases undisturbed by high thermal energy. These experimental observations are resembling with the manifestation of contrasting magnetic phases formation shown in the MCS study in this paper. We surmise that OMC-induced exchange coupling between two FM electrodes led to a voltage-induced anisotropy. We do not claim our hypothesis to be complete at this point, and further research and experimental studies are in order. In our future work, we also focus on experimental studies to understand the impact of multiple magnetic phases on the ferromagnetic electrodes and MTJMSD transport properties. We are initiating experimental studies with different types of molecules and magnetic metal electrodes to investigate promising combinations where molecules will not produce strong coupling between two metal electrodes. With weak molecule-induced exchange coupling, two ferromagnetic electrodes are expected to switch between parallel and antiparallel states using external field or spin torque effect. In the future Monte Carlo simulations, we will also focus on perpendicular magnetic anisotropy (PMA). PMA is expected to yield different equilibrium phases on the electrode and influence overall MTJMSD transport. MTJMSD may be adopted in various proposed configurations in prior literature^50^ to investigate STT-RAM.

Our Monte Carlo study is not periodical. Therefore, there will be size effects affecting the domain formation. We presently do not understand how increasing MTJMSD size for different anisotropy magnitude will impact MTJMSD properties. In future work, we plan to investigate the effect of increasing MTJMSD size for different strengths of unidirectional anisotropy.

## Supplementary Information

Supplementary Figures.

## Data Availability

The data supporting this study’s findings are available within the article [and its Supplementary Material]. The additional data supporting this study's findings are available from the corresponding author upon reasonable request.
