# Supplementary material for: Easy axis anisotropy creating high contrast magnetic zones on magnetic tunnel junctions based molecular spintronics devices (MTJMSD)
Source: Sci Rep. 2022 Apr 6;12:5721. doi: 10.1038/s41598-022-09321-7 (PMC8986785; doi:10.1038/s41598-022-09321-7)
Supplement: Supplementary file 1 — Supplementary Figures. [file 41598_2022_9321_MOESM1_ESM.docx]

**Supplementary Materials to**

**Easy Axis Anisotropy Creating High Contrast Magnetic Zones on Magnetic Tunnel Junctions Based Molecular Spintronics Devices (MTJMSD)**

*Bishnu R. Dahal^1^, Marzieh Savadkoohi^1^, Andrew Grizzle^1^, Christopher D’Angelo^1^, Vincent Lamberti^2^, and Pawan Tyagi^1*^*

*^1^Center for Nanotechnology Research and Education, Mechanical Engineering, University of the District of Columbia, Washington DC-20008, USA*

*^2^Y-12 National Security Complex, 301 Bear Creek Rd, Oak Ridge, TN 37830*

Corresponding Author Email: [*ptyagi@udc.edu*](mailto:ptyagi@udc.edu)

1. **Prior experimental data showing OMC paramagnetic molecular channels impacting ~7000 MTJs’ magnetic properties.**

**Figure S1**: (a) Magnetization vs magnetic field study of a Co/NiFe/AlOx/NIFe MTJ before after hosting OMCs to become MTJMSD; inset graph shows plot of *χ^-1^* vs. *T*. Tunnel junction with Pd and (b) top NiFe FM and (c) bottom Co/NiFe showing opposite response from OMCs . FMR study of Co/NiFe/AlOx/NiFe MTJ with (d) 2 nm AlOx and (e) 4 nm AlOx before and after OMCs interaction. (f) Topography and (g) MFM image of Co/NiFe/AlOx/NiFe MTJ based MTJMSD. (g) Green box shows OMC impacted MTJ lost magnetic contrast due to the OMC induced antiferromagnetic coupling. (g) Red color box show MTJ not impacted by OMC and showing typical strong magnetic contrast due to independent top and bottom ferromagnetic electrodes. (g) panel also showed molecule attachment process worked on ~75% MTJ. In general most of the MFM scans did not show any MTJ with high magnetic contrast suggesting OMC treatment impacted > 90% MTJs. Figure is adopted from Ref:25.


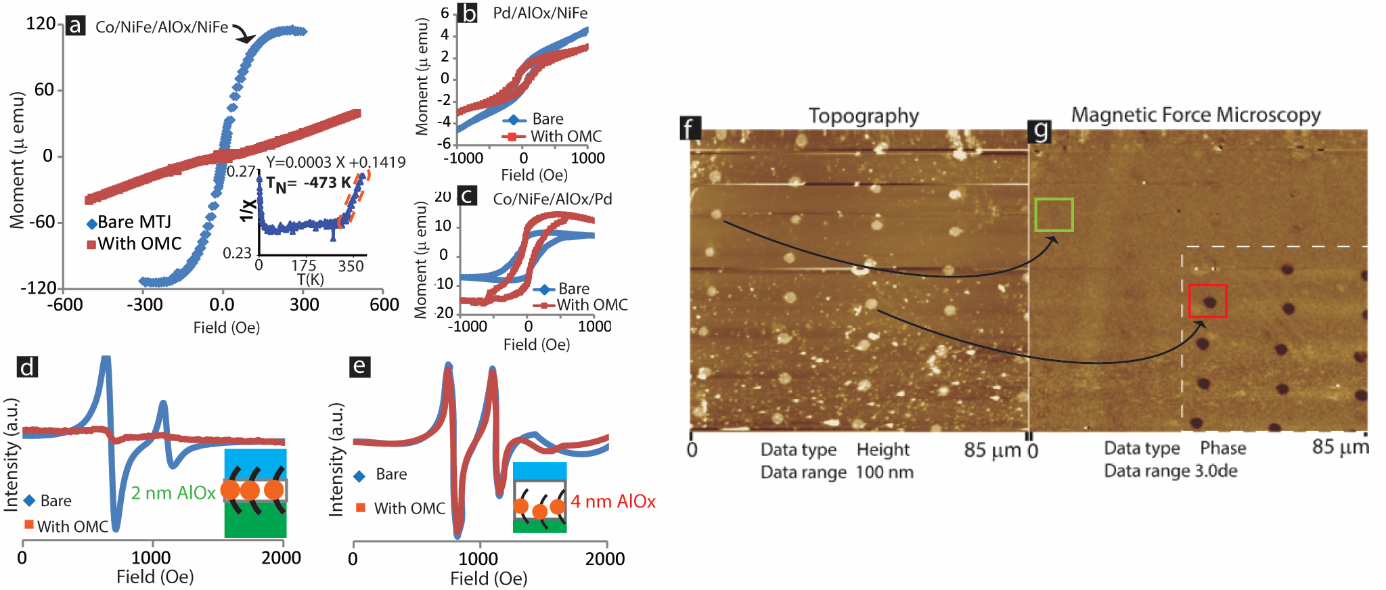


1. **3-D Lattice Model**


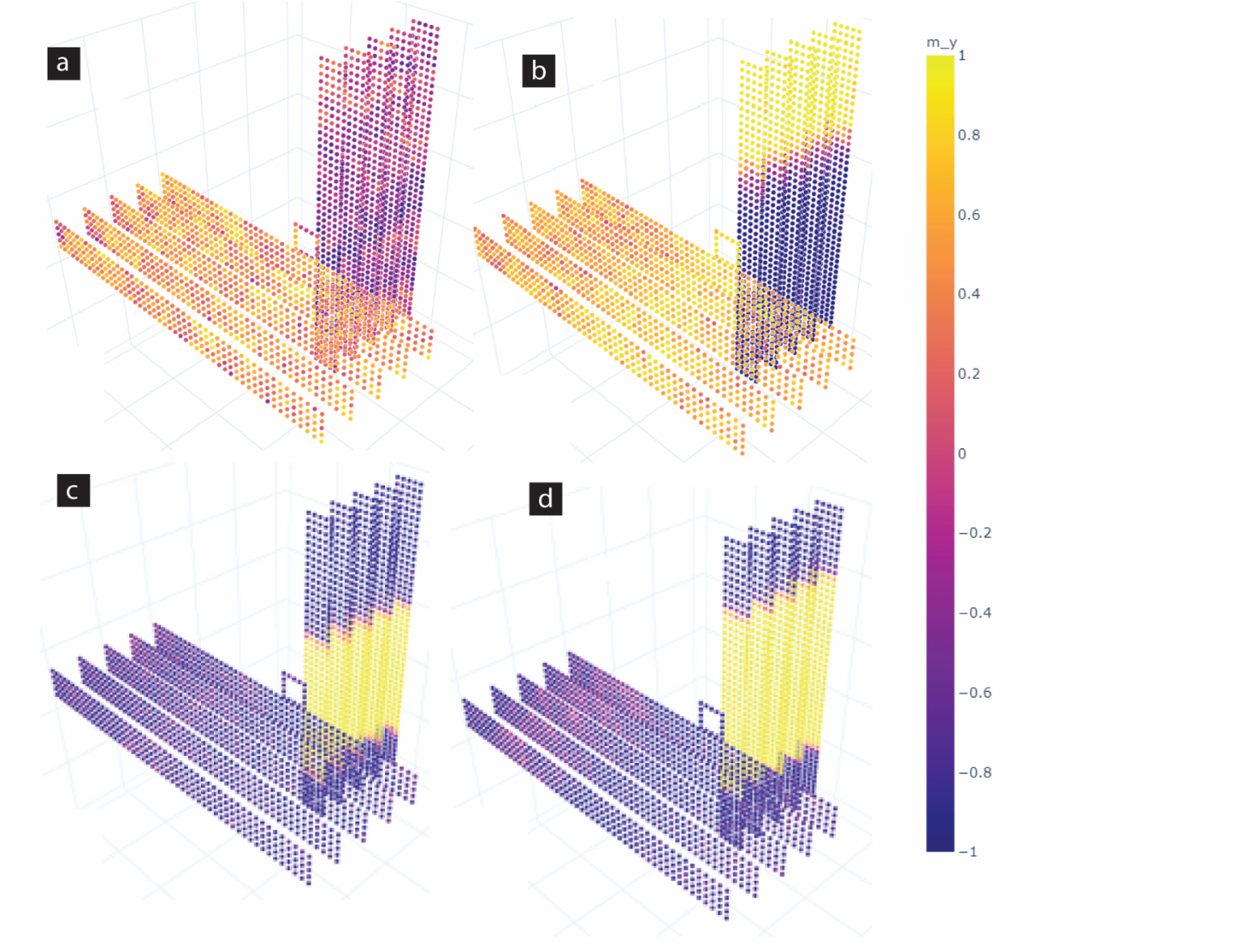


**Figure S2**: Simulated 3D lattice model of the MTJMSD measured at $kT$= 0.1 for (a) $A_{LY}$= 0, (b) $A_{LY}$ = 0.1, (C) $A_{ALY}$ = 0.4, and (d) $A_{LY}$ = 1.

1. **3-D Plot**


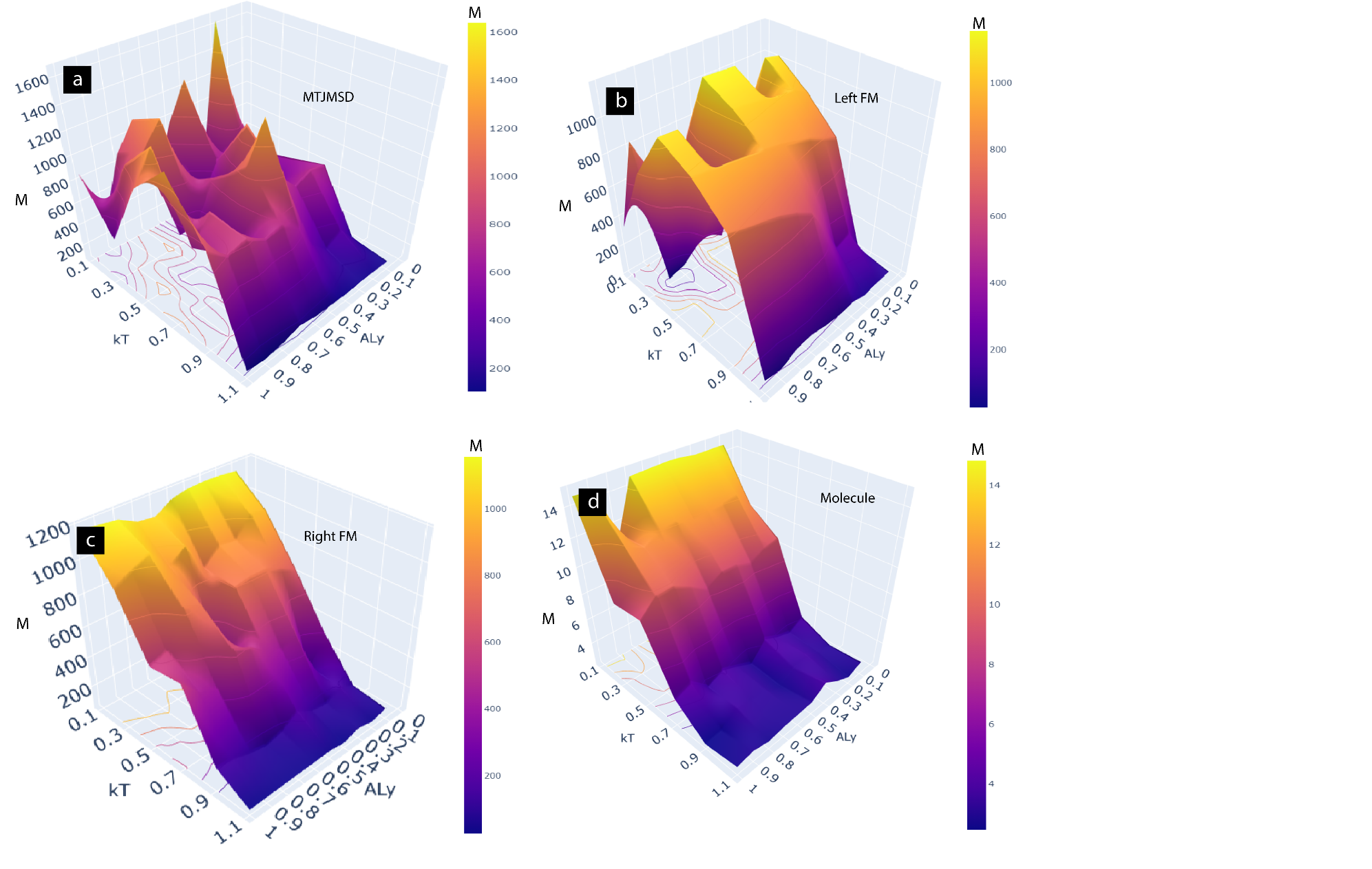


**Figure S3**: Magnetic moment of (a) MTJMSD, (b) Left FM electrode, (c) Right FM electrode, and (d) paramagnetic Molecules as a function of in-plane anisotropy and thermal energy ($kT$).

1. **Magnetic moment as function of in-plane anisotropy at constant thermal energy (second Trial)**


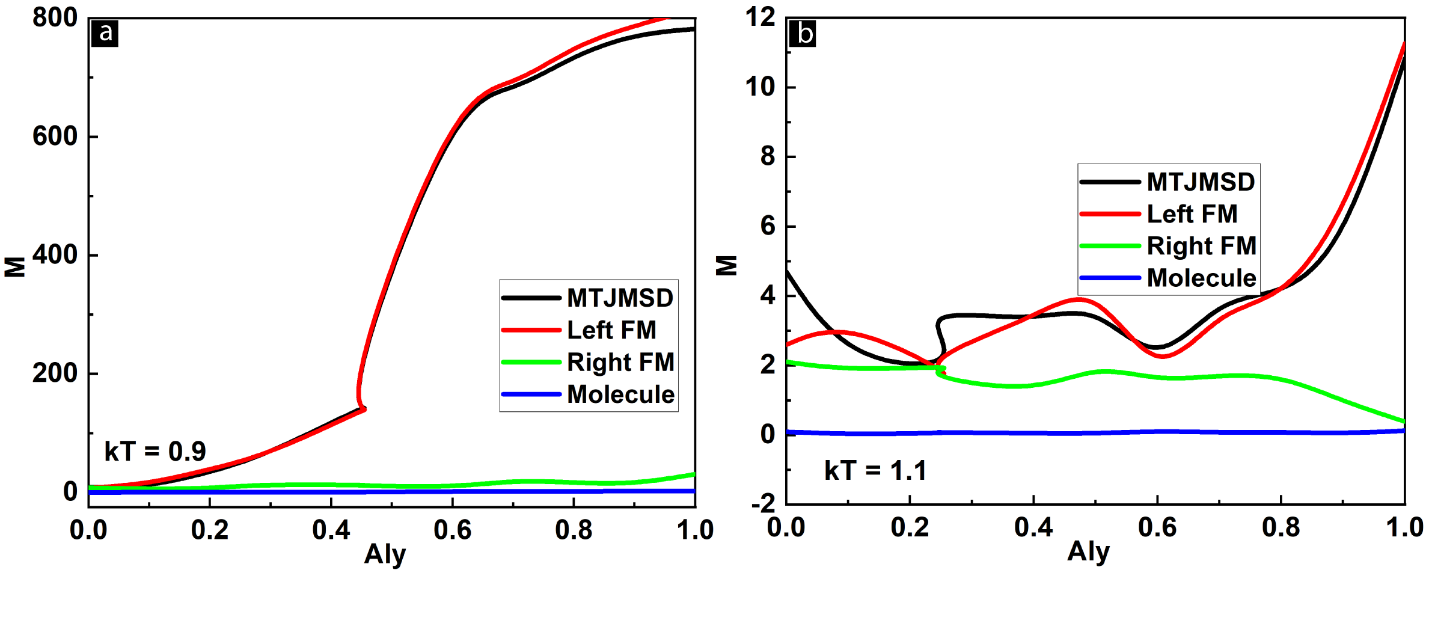
 **Figure S4**: Anisotropy dependence of magnetic moment obtained from continuous measurements: (a) 𝑘𝑇 = 0.9 and (b) 𝑘𝑇 = 1.1

5. Increasing easy-axis anisotropy produced sharper domain boundary between high contrast magnetic zones. Multiple magnetic domain boundaries started appearing with increasing easy-axis anisotropy (**Figure S5, supplementary section**). The graph below shows the SC factor as a function of electrode length for different easy-axis anisotropy strengths. Data is taken along the length from the SC spatial panels in Figure 4.


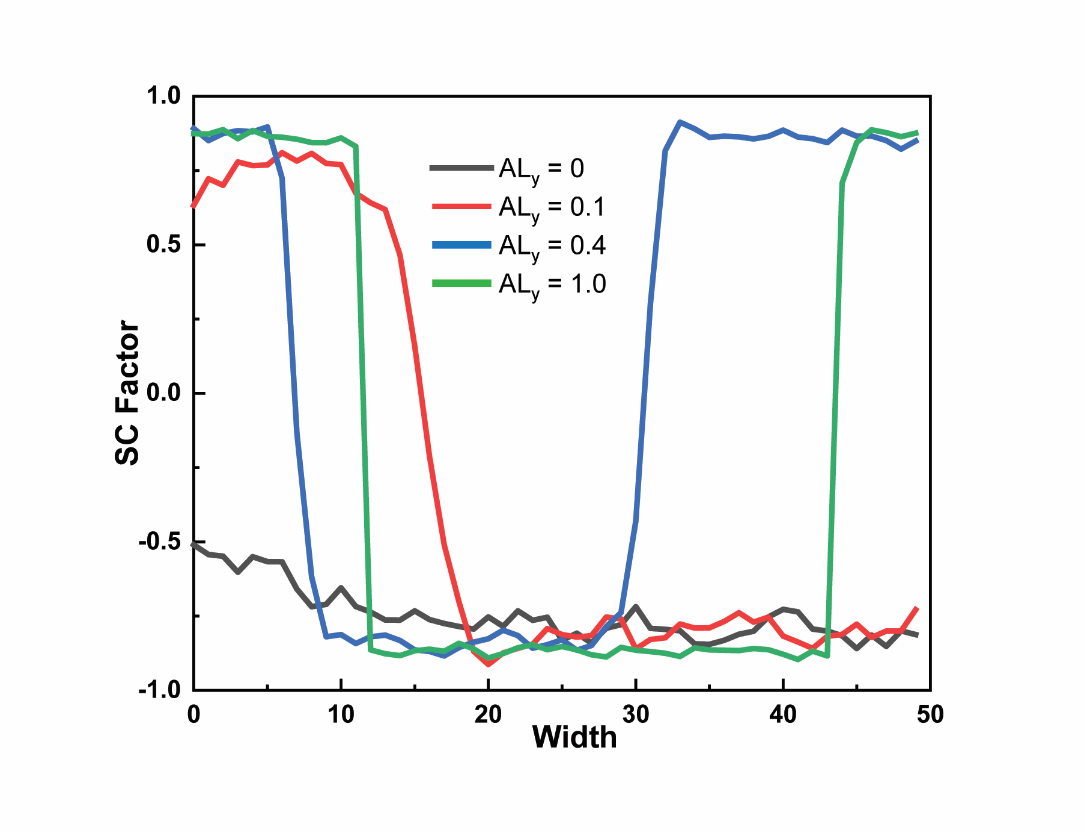


**Figure S5**: Easy axis anisotropy governing the width of domains represented in Figure 4 𝑘𝑇 = 0.1.
